# Medical Reform and Private Instructors

**Published:** 1852-07

**Authors:** 


*Medical Reform and Private Instructors.*—In view of the clamor for various plans embodying supposed improvements in medical education, usually presented under the *ad captandum* title of *Medical Reform*, we have, on several occasions, endeavored to call attention to the fact, that the most important and efficient step, in behalf of the elevation of the character of the profession, is to be taken by its members individually; in other words, that the measure of so called reform which is most needed at the present time, applies directly to every practitioner who receives into his office private pupils. The burthen of the prevailing complaints of the system of medical instruction, in this country, is directed toward the colleges. Without contending that these institutions are faultless, it is to our mind sufficiently clear, that far greater blame, (if blame is to be attached any where,) lies with the private teacher, who admits pupils into his office wholly unprepared to enter on the study of medicine, and leaves him to steer his way without rudder or pilot, in the intervals of his collegiate courses. In the following extract from an article on medical reform, contained in a late number of the Transylvania Medical Journal, this topic is discussed in a manner which will, we think, commend itself to the common sense of the medical reader:

When Lycurgus was consulting with the people in reference to the reform of the State of Sparta, one advised the establishment of absolute popular equality: Lycurgus replied to him, "Sir, begin it in your own house." In like manner we would say to every medical man, who is really anxious for medieal reform, "Sir, begin it in your own office." And we do not say it, as did Lycurgus, in the spirit of retort, with a view of indicating the impracticability of the thing, but in all sincerity, because it is in truth and in fact in the office of the private practitioner that the desired reform can be most appropriately and effectually initiated. Medical students, almost without exception, enter upon the study of the profession under the immediate advice and direction of some private practitioner, who is regarded as their personal friend or the friend of the family, or who is selected on some account or other as a suitable person from whom to seek advice as to the propriety of engaging in the study of medicine, as well as guidance in its prosecution. They reach the schools usually after they have been for some time studying under the direction of their private preceptors, and, of course, after their minds are fully made up as to the propriety of their choice of pursuit. At this late period it would be utterly idle in the professors of the Schools to attempt to change the determination of such as may seem to them to have acted unwisely in conceiving themselves prepared to enter upon the study of their choice; because any such advice as might look to the abandonment of their purpose, would be not only unthankfully received by the pupil, but justly regarded as an improper, if not impertinent, interference with the prerogatives of the private preceptor. Medical colleges are public institutions, chartered by the proper authorities for the purpose of affording facilities for the acquisition of medical education, and not for the purpose of deciding who shall or who shall not study medicine. Their managers should consider themselves bound, it seems to us, to receive the pupil of any respectable practitioner, who may wish to prosecute the study of the profession with a view of obtaining the degree which the schools alone are authorized to confer. The duties of their teachers consist in a faithful endeavor to present to the consideration of the pupils the ascertained and reliable knowledge of the profession, and an earnest effort to impress it as permanently as possible upon their minds, and in the honest examination of the candidates, and a just decision upon the propriety of admitting them to the honors of the Doctorate. They have nothing to do, in their capacity of public teachers, with the determination of the choice of pursuit by the various pupils who resort to their lecture rooms. This has been decided, as it should be, by the private preceptor, and with him, and him alone, rests the responsibility which depends upon the issue. Were the schools to undertake the ungracious task of changing the purpose of pupils who resort to their lecture rooms, under the advice of their private preceptors, there would be one universal outbreak of indignation, on the part of practitioners, and the schools would be justly denounced as arrogant monopolies. For ourself, having the utmost confidence in the general intelligence and honesty of our professional brethren, we are more than willing to concede to them the exclusive right to decide upon the fitness or unfitness of all applicants to engage in the study of medicine, and, when they come to the schools with such endorsement, no inquiry should be made as to the correctness or incorrectness of this primary decision. We are unwilling that the responsibility of advising men to engage in the study of medicine should be shifted from the private preceptors, where it properly belongs, to the public teachers, who, as such, should have nothing to do with it. On the contrary, we would say to the practitioners of medicine throughout the Union, upon you, gentlemen, must rest all the responsibility connected with the admission of pupils to the study of the profession. Your advice is first sought, and by it the course of the pupil generally determined. This first step is one of paramount importance, as it involves not only the question of preparatory education, but also of moral, physical and intellectual qualification for the responsible ministry of our noble profession. The time for making the effort to change the purpose of such as are not possessed of the requisite qualification, is when the purpose is first formed, and before the personal pride and interest of the novitiate have become involved.

But, we may be here met with the suggestion that these errors can be corrected by the refusal of the schools to admit incompetent or unsuitable persons to the honor of the doctorate. It may be said, it matters but little who is received into the office of the private practitioner, since he has the right to look to the schools for a correct decision upon the merits of the candidate for graduation. To this suggestion, however, there are two conclusive replies, to say nothing of others. In the first place, it is by no means certain, that the pupil will not engage in practice before he attempts to graduate, and thus deprive the schools of the opportunity of passing judgment upon him. And, in the second place, the rejection, as it is technically termed, does not drive the candidate from the profession, but rather forces him, in self-defence, either to engage in practice, or to persevere until he can, charge his memory by a system of grinding, with a sufficient knowledge of details to enable him to obtain, from some source or other, the desired degree. So that, as a general fact, the mere admission of an unsuitable, unqualified person into the doctor's office as a pupil, decides the question of his ultimate introduction into the ranks of the profession; and, in this view of the matter, how important does it become that medical practitioners should exercise a judicious discretion in receiving pupils into their offices? How indispensable, indeed, is the exercise of such discretion to anything like a successful, safe or just scheme of medical reform? We say again, then, to our brethren, who are clamorous for reform — "Gentlemen, begin it in your own offices."

